# Iodanyl Radical Catalysis

**DOI:** 10.1021/acs.accounts.3c00231

**Published:** 2023-07-06

**Authors:** Asim Maity, Brandon L. Frey, David C. Powers

**Affiliations:** Texas A&M University, College Station, Texas 77843, United States

## Abstract

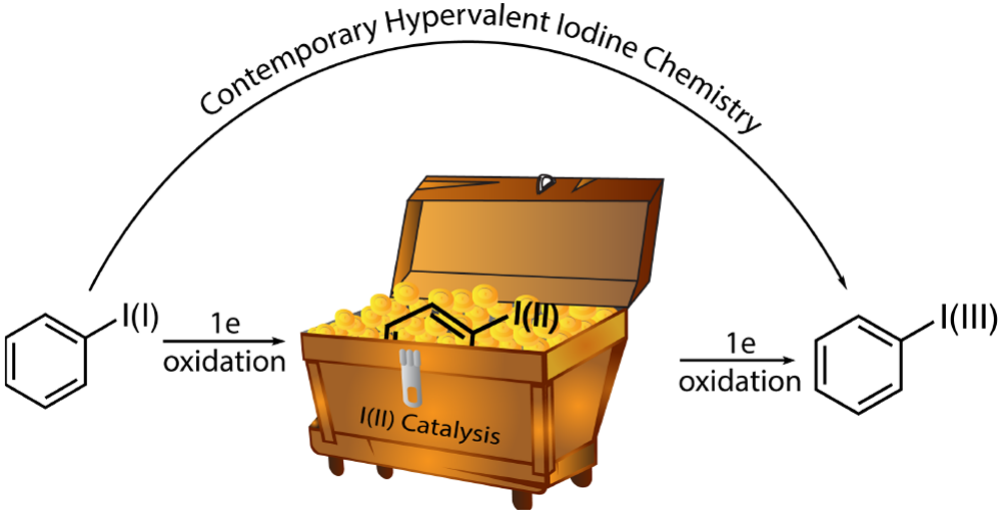

Hypervalent iodine reagents find application as selective chemical oxidants in a diverse array of oxidative transformations. The utility of these reagents is often ascribed to (1) the proclivity to engage being selective two-electron redox transformations; (2) facile ligand exchange at the three-centered, four-electron (3c–4e) hypervalent iodine–ligand (I–X) bonds; and (3) the hypernucleofugacity of aryl iodides. One-electron redox and iodine radical chemistry is well-precedented in the context of inorganic hypervalent iodine chemistry—for example, in the iodide–triiodide couple that drives dye-sensitized solar cells. In contrast, organic hypervalent iodine chemistry has historically been dominated by the two-electron I(I)/I(III) and I(III)/I(V) redox couples, which results from intrinsic instability of the intervening odd-electron species. Transient iodanyl radicals (i.e., formally I(II) species), generated by reductive activation of hypervalent I–X bonds, have recently gained attention as potential intermediates in hypervalent iodine chemistry. Importantly, these open-shell intermediates are typically generated by activation of stoichiometric hypervalent iodine reagents, and the role of the iodanyl radical in substrate functionalization and catalysis is largely unknown.

Our group has been interested in advancing the chemistry of iodanyl radicals as intermediates in the sustainable synthesis of hypervalent I(III) and I(V) compounds and as novel platforms for substrate activation at open-shell main-group intermediates. In 2018, we disclosed the first example of aerobic hypervalent iodine catalysis by intercepting reactive intermediates in aldehyde autoxidation chemistry. While we initially hypothesized that the observed oxidation was accomplished by aerobically generated peracids via a two-electron I(I)-to-I(III) oxidation reaction, detailed mechanistic studies revealed the critical role of acetate-stabilized iodanyl radical intermediates. We subsequently leveraged these mechanistic insights to develop hypervalent iodine electrocatalysis. Our studies resulted in the identification of new catalyst design principles that give rise to highly efficient organoiodide electrocatalysts that operate at modest applied potentials. These advances addressed classical challenges in hypervalent iodine electrocatalysis related to the need for high applied potentials and high catalyst loadings. In some cases, we were able to isolate the anodically generated iodanyl radical intermediates, which allowed direct interrogation of the elementary chemical reactions characteristic of iodanyl radicals. Both substrate activation via bidirectional proton-coupled electron transfer (PCET) reactions at I(II) intermediates and disproportionation reactions of I(II) species to generate I(III) compounds have been experimentally validated.

This Account discusses the emerging synthetic and catalytic chemistry of iodanyl radicals. Results from our group have demonstrated that these open-shell species can play a critical role in sustainable synthesis of hypervalent iodine reagents and play a heretofore unappreciated role in catalysis. Realization of I(I)/I(II) catalytic cycles as a mechanistic alternative to canonical two-electron iodine redox chemistry promises to open new avenues to application of organoiodides in catalysis.

## Key References

- MaityA.; HyunS. M.; PowersD. C.Oxidase Catalysis via Aerobically Generated Hypervalent Iodine Intermediates. Nat. Chem.2018, 10, 200–204.29359760 10.1038/nchem.2873(1) This work reported aldehyde autoxidation-promoted aerobic synthesis of hypervalent iodine compounds and aryl iodide-catalyzed aerobic oxidation chemistry.
- HyunS.-M.; YuanM.; MaityA.; GutierrezO.; PowersD. C.The Role of Iodanyl Radicals as Critical Chain Carriers in Aerobic Hypervalent Iodine Chemistry. Chem2019, 5, 2388–2404.(2) This work investigated the mechanism of aldehyde autoxidation-promoted aerobic hypervalent iodine chemistry and implicated the intermediacy of transient iodanyl radicals.
- MaityA.; FreyB. L.; HoskinsonN. D.; PowersD. C.Electrocatalytic C–N Coupling via Anodically Generated Hypervalent Iodine Intermediates. J. Am. Chem. Soc.2020, 142, 4990–4995.32129617 10.1021/jacs.9b13918(3) This work targeted anodically generated iodanyl radicals to extend sustainable hypervalent iodine catalysis into electrochemical contexts.
- FreyB. L.; FigginsM. T.; Van TriesteG. P.III; CarmieliR.; PowersD. C.Iodine–Iodine Cooperation Enables Metal-Free C–N Bond-Forming Electrocatalysis via Isolable Iodanyl Radicals. J. Am. Chem. Soc.2022, 144, 13913–13919.35856717 10.1021/jacs.2c05562PMC10251780(4) This work reported cooperative I–I bonding as a design principle to engender highly reversible electrochemistry and efficient C–N coupling catalysts via isolable iodanyl radical intermediates.

## 1 Introduction

The demand for sustainable synthetic chemistry requires the invention of new methods that rely on readily available renewable chemical resources and that do not impose the generation of significant chemical waste streams. In the context of oxidation chemistry, which is often required to convert commodities to fine chemicals, atmospheric dioxygen (O_2_) and solar-generated electron holes are among the few potential sustainable terminal oxidants available on scale.

Application of either O_2_ or anodic oxidation events in synthetic chemistry must confront a common challenge: How does one selectively couple the one-electron chemistry characteristic of the triplet ground state of O_2_ or the interfacial electron transfer events that underpin electrochemistry, to achieve selective multielectron bond-making and -breaking events? One commonly employed strategy relies on redox mediators (or catalysts), which are small molecules that engage in well-defined redox chemistry and can aggregate the single-electron steps of sustainable oxidation with the multielectron reactions typical of organic synthesis. In this context, aerobic or electrochemical oxidation of hydroquinone to quinone, which aggregates two electron–hole equivalents and can engage in substrate activation chemistry, is illustrative.^5^

**Figure 1:**
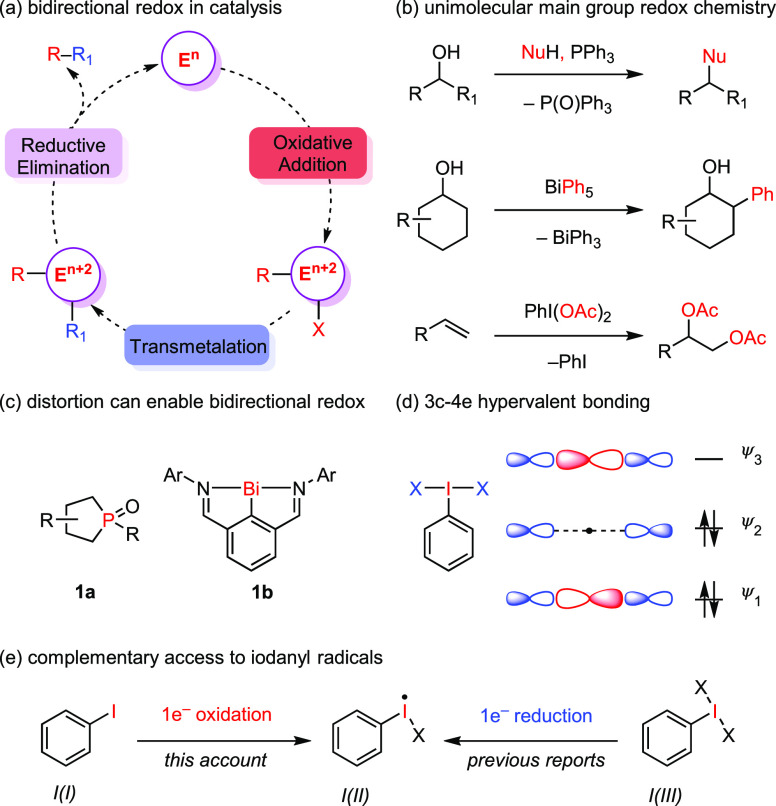
(a) A generic catalytic cycle for cross-coupling relies on ligand exchange and bidirectional two-electron redox steps that are common of second- and third-row transition metal ions but uncommon for main group elements. (b) Examples of stoichiometric main group redox chemistry with phosphorus, bismuth, and iodine. (c) Geometric distortion can enable bidirectional redox chemistry, and thus catalysis, at heavy main group elements. (d) Three-centered, four-electron (3c–4e) bonding model used to describe hypervalent bonding in I(III) compounds. (e) While the previous reports were based on reductive generation of iodanyl radicals, this account describes one-electron oxidation of aryl iodides to afford iodanyl radicals.

Biology has evolved elaborate mechanisms for sustainable redox chemistry through exquisitely choreographed delivery of proton and electron equivalents to leverage the one-electron chemistry of earth-abundant first-row metal ions to achieve multielectron chemistry.^6,7^ In comparison, second- and third-row metals are more often deployed in chemosynthetic oxidation reactions, which is a reflection of the facile bidirectional redox chemistry displayed by many transition metal ions (d-orbital splitting ≤ 4 eV)^8^ and the intrinsic two-electron redox couples that are characteristic of these transition metals (Figure 1a).^9^ The instability of odd-electron states in the heavier transition metals is often attributed to the larger orbital amplitude of these ions, which results in facile bimolecular disproportionation reactions.

In similarity to transition metals, many heavy main group elements can access multiple stable oxidation states and have emerged as potential platforms for metal-free redox catalysis. As with second- and third-row transition metal ions, the diffuse frontier orbitals of heavy main group elements tend to result in rapid disproportionation chemistry of odd-electron species and thus the appearance of selective two-electron redox couples. In contrast to the relatively close orbital spacing of transition metal ions, the relatively large energy spacing of the valence s and p orbitals of these elements (typically >4 eV) often prevents facile bidirectional redox chemistry. For example, oxidative addition reactions at low valent Mg,^10^ Al,^11^ Si,^12^ Ge,^13^ and Sn^14,15^ are common, but reductive elimination from high valent species of these metals is often found challenging to achieve.^16,17^ Similarly, reductive elimination from high valent Bi, S, and I is facile, but corresponding oxidative addition is difficult.^18−21^ As a result, heavy main group reagents are often encountered as stoichiometric reagents in synthetic chemistry but are relatively rarely implemented as catalysts (Figure 1b).^22−24^ Geometrical distortion of the coordination geometry of heavy main group elements, such as is observed in P(III) and Bi(III) complexes **1a** and **1b**, respectively, can narrow the gap between the frontier orbitals and give rise to more facile redox interconversion (Figure 1c). These strategies are currently gaining momentum as an approach to elicit metal-free multielectron redox catalysis.^25,26^

We were attracted to the two-electron redox proclivity of heavy main-group elements as a platform to marry the one-electron processes characteristic of sustainable oxidation chemistry with selective substrate functionalization. In particular, we have pursued opportunities in sustainable hypervalent iodine chemistry because (1) iodine displays a number of stable oxidation states, and oxidation of organoiodides by two- or four-electrons results in hypervalent I(III) and I(V) compounds, respectively; (2) unidirectional reduction from I(V) to I(III) or I(III) to I(I) can be coupled to a wide variety of substrate oxidation reactions including asymmetric reactions;^27−31^ and (3) hypervalent iodine compounds readily engage in elementary reaction steps such as ligand exchange and reductive elimination by virtue of the hypernucleofugacity of aryl iodides and the lability of the 3c–4e hypervalent bonds (Figure 1d).^32,33^ The structure, bonding, and elementary reaction chemistry of hypervalent iodine(III) and I(V) reagents has been extensively reviewed.^29−31,34^

An intrinsic drawback of utilizing unidirectional redox chemistry is the obligate use of stoichiometric loadings of hypervalent iodine reagents (and the attendant formation of stoichiometric waste streams). As we began our work, progress toward catalytic hypervalent iodine protocols had begun to emerge;^31,35,36^ however, widespread deployment of hypervalent iodine catalysis continues to confront challenges in selective oxidation of aryl iodides over oxidatively labile substrates present in the reaction.

Our program in sustainable hypervalent iodine chemistry was motivated by the hypothesis that development of aerobic and electrochemical methods to generate hypervalent iodine reagents would provide the platform to couple environmentally benign oxidants with a diverse array of substrate activation mechanisms. We initiated our efforts by developing aerobic methods to oxidize aryl iodides to hypervalent I(III) compounds. Detailed mechanistic investigations of the resulting methods revealed the potential that one-electron processes, not two electron elementary steps, were operating and indicating an unanticipated role of iodanyl radicals. This discovery first stimulated the development of new electrocatalytic C–N bond-forming reactions and subsequently led to the realization that iodanyl radicals themselves may be competent in substrate activation chemistry without further oxidation to discrete hypervalent I(III) compounds. While iodanyl radicals had been proposed as intermediates in the reductive activation of stoichiometric hypervalent I(III) reagents,^37^ the potential role of I(II) species in the synthesis of hypervalent iodine compounds and in catalytic applications had not been previously described (Figure 1e). This Account describes the development of iodanyl radical catalysis and seeks to identify opportunities available to controlling the chemistry of iodanyl radicals.

## 2 Aerobic Hypervalent Iodine Chemistry and Catalysis

**Figure 2:**
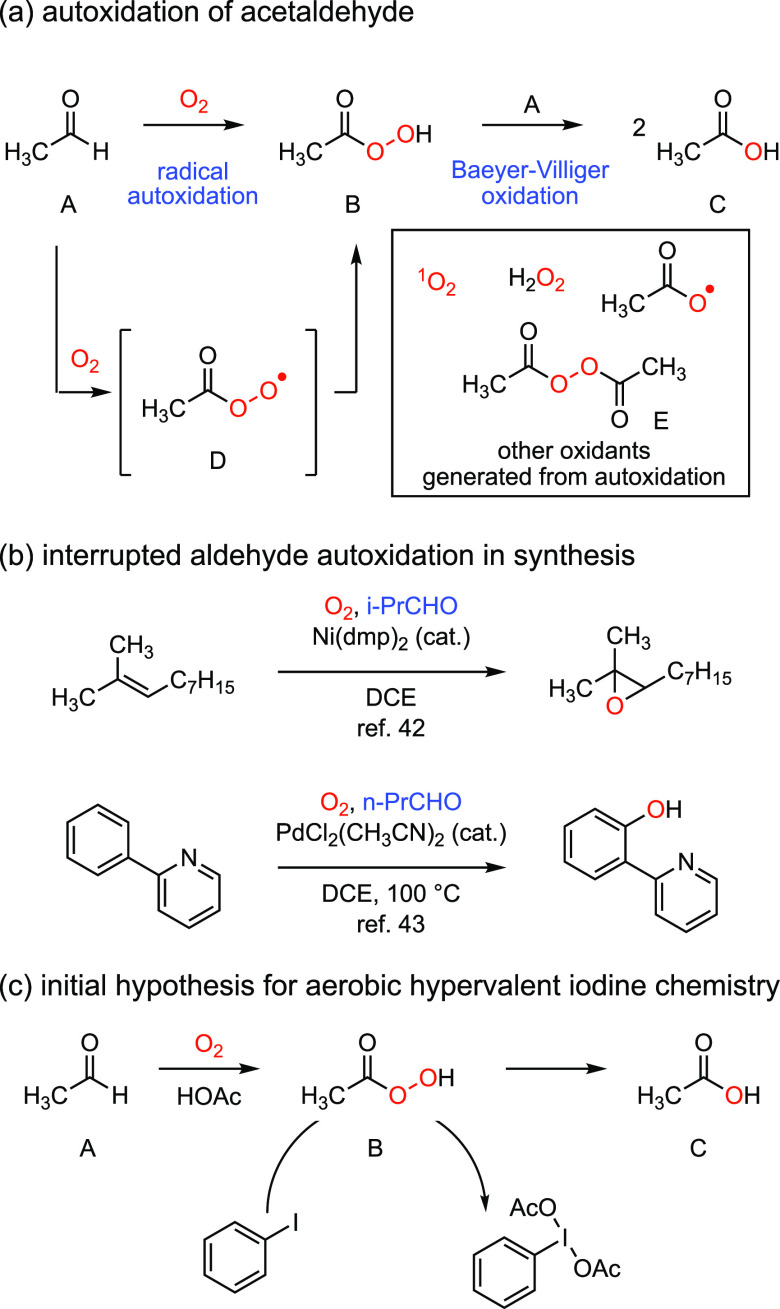
(a) Aerobic oxidation of acetaldehyde proceeds via radical autoxidation to generate peracetic acid followed by nonradical Baeyer–Villiger chemistry to generate acetic acid. A variety of off-path reactive oxygen species (ROSs) can be generated during autoxidation. (b) Peracid and peroxy radical intermediates generated during aldehyde autoxidation have been intercepted for transition metal-catalyzed oxygenation reactions. (c) We initially targeted aerobic synthesis of hypervalent iodine compounds based on the hypothesis that peracid intermediates could be intercepted by aryl iodides. Ni(dmp): bis[1,3-bis(*p*-methoxyphenyl)-1,3-propanedionato] nickel(II).

Direct oxidation of PhI with 1/2 O_2_ to generate PhIO is thermodynamically favorable but has not been experimentally observed, presumably due to kinetic barriers implied by the triplet ground state of O_2_.^38^ As we considered strategies to marry O_2_ reduction with aryl iodide oxidation, we were attracted to the potential to intercept oxidizing intermediates generated during aldehyde autoxidation. Aldehyde autoxidation proceeds via a radical chain mechanism to generate peracid intermediates, which subsequently engage in Baeyer–Villiger chemistry to afford carboxylic acids (Figure 2a).^39,40^ Inspired by reports of oxygen-atom transfer from reactive autoxidation intermediates (Figure 2b),^41−44^ we envisioned that aerobic synthesis of hypervalent iodine compounds could be achieved by intercepting aerobically generated peracid intermediates with aryl iodides (Figure 2c).^45^

**Figure 3:**
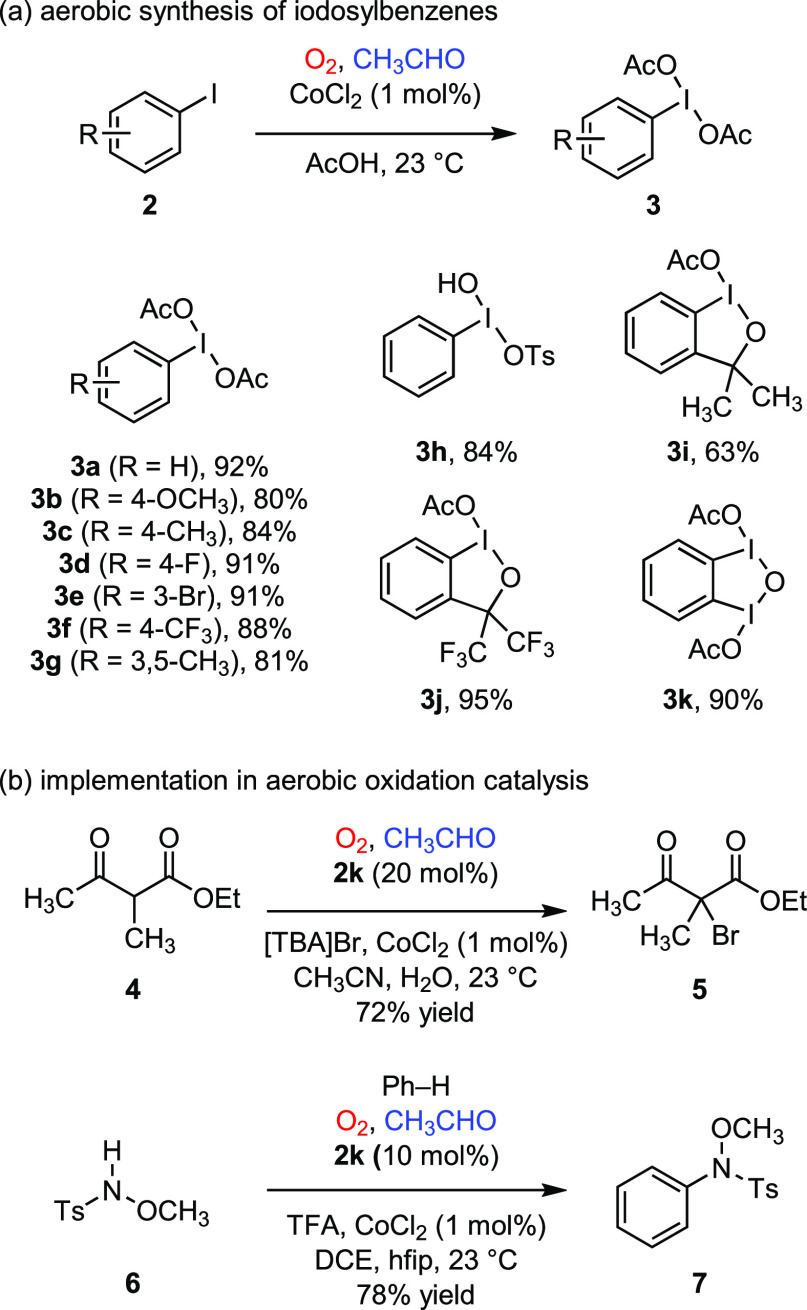
(a) Aerobic synthesis of I(III) reagents via interrupted aldehyde autoxidation. (b) Implementation of aerobic hypervalent iodine catalysis in bromination and metal-free intermolecular C–H amination. [TBA], *tetra*-butyl ammonium; TFA, trifluoroacetic acid; hfip, 1,1,1,3,3,3-hexafluoroisopropanol; DCE, 1,2-dichloroethane.

We rapidly reduced this hypothetical scheme to practice: Exposure of acetic acid or 1,2-dichloroethane (DCE) solutions of iodobenzene (PhI) to acetaldehyde under an atmosphere of O_2_ resulted in the formation of I(III)-reagent diacetoxyiodobenzene (**3a**, PhI(OAc)_2_).^1^ The yield and kinetics of the aerobic oxidation reaction were found to be more reproducible when 1 mol % CoCl_2_ was added to the reaction, which presumably facilitates the initiation of the aldehyde autoxidation chain reaction via the formation of Co superoxide adducts.^39,46^ The developed conditions provided efficient access to a family of hypervalent iodine reagents that are commonly encountered in synthetic chemistry (Figure 3a): 4-Substituted aryl iodides with electron-donating and -withdrawing groups engage in efficient oxidation to afford the corresponding I(III) compounds (**3b**–**3h**); benziodoxole-based hypervalent iodine reagents (**3i**, **3j**) were readily accessed; and 1,2-diiodobenzene (**2k**) was oxidized to the corresponding bis-ester (**3k**) in high yield without the generation of potential mixed-valent hypervalent iodine byproducts.

The developed aerobic oxidation conditions enabled a variety of hypervalent iodine-catalyzed aerobic oxidation reactions.^1,47^ In particular, the interrupted aldehyde autoxidation conditions enabled aryl iodide-catalyzed bromination chemistry—for example, aerobic oxidation reaction with β-ketoester **4** in the presence of [TBA]Br afforded brominated ethylacetoacetate derivative **5**—and C–H amination chemistry—for example, the formation benzenesulfonamide derivative (**7**) from benzene and sulfonamide (**6**, Figure 3b).^1^ In each of these reactions, O_2_ was employed as the terminal oxidant, and acetic acid was the only stoichiometric byproduct. Application of these conditions to olefin functionalization chemistry, which represents a large and synthetically important family of hypervalent iodine promoted transformations, was unsuccessful, presumably due to competitive olefin oxidation by reactive oxygen species generated during aldehyde autoxidation.

## 3 Iodanyl Radicals in Aerobic Hypervalent Iodine Catalysis

**Figure 4:**
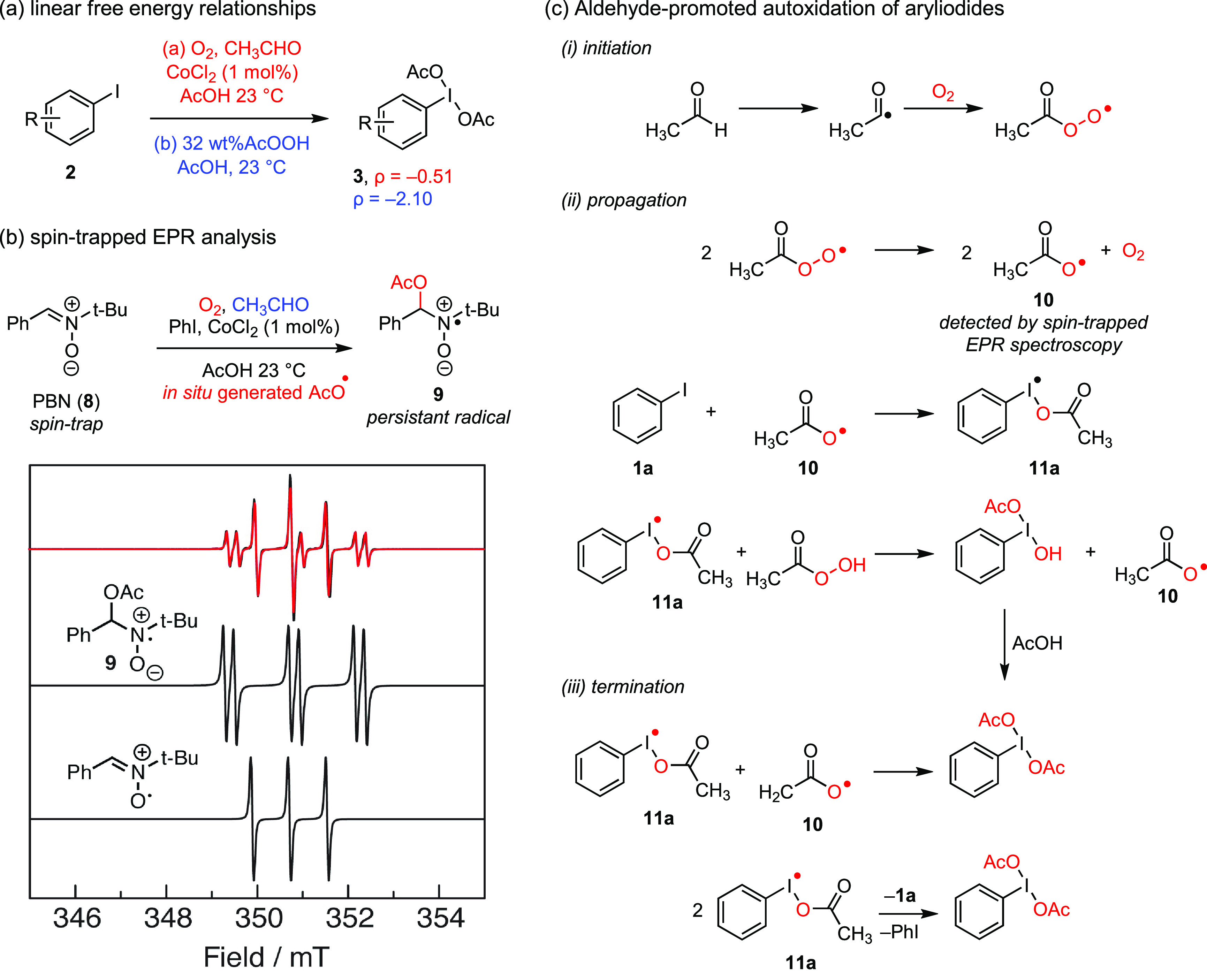
(a) Comparison of linear free energy relationships for the aerobic and peracid-based hypervalent iodine syntheses. (b) Spin-trapped EPR analysis of aldehyde-promoted PhI oxidation. The experimentally obtained EPR spectrum (**—**) overlays with an admixture of the spectra of the radical generated by one-electron oxidation of **8** and the acetoxy radical adduct of PBN (**9**). (c) Proposed radical chain mechanism of aldehyde-promoted aerobic oxidation of aryl iodides (**2a**) via acetoxy radical **11a**. PBN: phenyl *N*-*t*-butylnitrone.

The development of aerobic hypervalent iodine chemistry was predicated on the hypothesis that aerobically generated peracid intermediates were responsible for aryl iodide oxidation (Figure 2c).^1^ With interest in developing more broadly applicable catalytic protocols (e.g., application of sustainable oxidation methods to olefin functionalization chemistry), we initiated a detailed investigation of the mechanism of aerobic oxidation and found that our initial hypothesis was incorrect and that open-shell iodine species are critical intermediates in the developed aerobic oxidation chemistry.^2^ The following lines of evidence were inconsistent with the original peracid hypothesis and instead pointed to an iodanyl radical-based autoxidation mechanism: First, Hammett analysis of a family of 4-substituted aryl iodides revealed different kinetic behavior for peracetic acid oxidation (ρ = −2.10, correlated with σ parameters) and our aerobic oxidation (ρ = −0.51, correlated with σ^+^ parameters, Figure 4a). This observation indicated that aldehyde-promoted aerobic oxidation and peracid-mediated oxidation do not proceed via the same rate-determining elementary step. Second, aryl iodides were found to be kinetic inhibitors of C–H oxidation of cyclohexane, ethylbenzene, and adamantane under aldehyde autoxidation conditions. This observation suggests that aryl iodides can engage the oxygen-centered radicals that are responsible for H-atom abstraction under these conditions. Third, spin-trapped EPR studies with PBN (**8**) resulted in detection of the acetoxy radical adduct of PBN (i.e., **9**) during aldehyde-promoted aerobic oxidation of iodobenzene (Figure 4b). Finally, Density Functional Theory (DFT) studies indicated a low barrier for the addition of carboxy radicals to aryl iodides. Together, these experimental and theoretical data pointed to a radical chain mechanism propagated by chain-carrying iodanyl radical intermediate **11a** (Figure 4c). Radical chain termination is proposed to happen either via combination of I(II) species (**11a**) with an acetoxy radical (**10**) or disproportionation of **11a** to afford PhI(OAc)_2_ and PhI.

The intermediacy of iodanyl radical intermediates in the autoxidation of aryl iodides has mechanistic echoes of phosphine autoxidation, which has been proposed to proceed through transient phosphanyl radicals (i.e., P(IV) species)^48,49^ and in the autoxidation of late metal alkyl complexes, such as Pd(bpy)Me_2_, which has been proposed to proceed via the intermediacy of transient Pd(III) intermediates.^50^ The common theme of these reactions is that the atom undergoing oxidation can expand its valence, and thus unlike organic autoxidation chemistry, which proceeds via H-atom abstraction and radical propagation steps, autoxidation at these heavier elements can proceed via radical addition reactions.

The realization of iodanyl radical intermediates was significant because it suggested a productive role for I(II) species in the synthesis of hypervalent iodine compounds. Previous reports of transient I(II) intermediates focused on the reductive generation of these species by I–X homolysis in ArIX_2_ compounds (Figure 1e).^37,51^ In addition, while reductively generated iodanyl radicals have been proposed in various photochemical and photoredox schemes, the role of the putative I(II) intermediates in substrate functionalization has largely not been evaluated (*vide infra*).

## 4 Iodanyl Radical Electrochemistry and Electrocatalysis

While interrupted aldehyde autoxidation chemistry enabled oxidation catalysis using aerobically generated hypervalent iodine intermediates, the obligate generation of reactive oxygen species in the autoxidation manifold resulted in difficulty applying the conditions to catalysis with oxidatively labile substrates, such as olefins. These limitations mandated the development of complementary methods for sustainable hypervalent iodine chemistry. Based on the mechanistic hypothesis that acetate-stabilized iodanyl radicals could be viable intermediates en route to aerobically generated hypervalent iodine species, we considered accessing a similar mechanistic paradigm electrochemically. Specifically, we considered that an acetoxy radical could be generated by interrupted Kolbe electrochemistry (i.e., anodic oxidation of acetate)^52^ and could provide access to hypervalent iodine electrochemistry analogous to the aforementioned aerobic oxidation chemistry.

**Figure 5:**
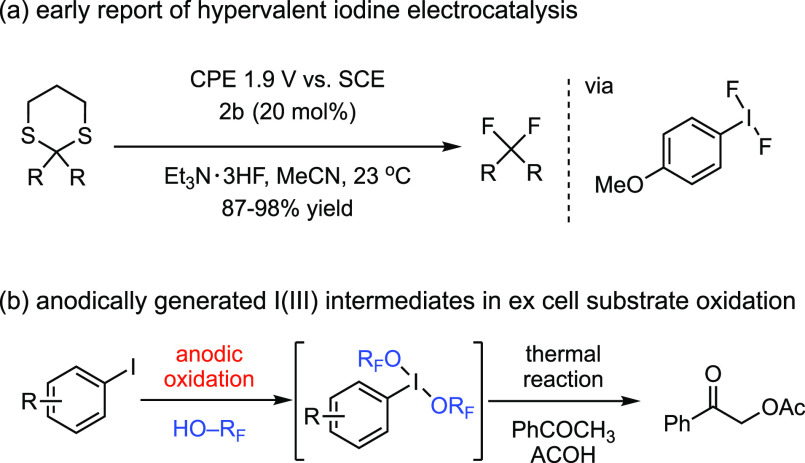
(a) Oxidatively resistant fluoride salts were key to achieving the first report of hypervalent iodine electrolysis. (b) Representative application of anodically generated hypervalent iodine intermediates in *ex cell* substrate oxidation reactions. HO–R_F_: hfip (1,1,1,3,3,3-hexafluoroisopropanol), TFE (2,2,2-trifluoroethanol).

At the outset of our studies, we were cognizant of the long-standing challenges to hypervalent iodine electrocatalysis, namely, the need for high applied potentials and the often irreversible electrochemistry of aryl iodides that can result in catalyst decomposition.^53^ These challenges have resulted in (1) few *bona fide* examples of electrocatalysis, with successes limited to oxidatively resistant fluoride reagents (i.e., catalysis via ArIF_2_, Figure 5a),^54^ and (2) *ex cell* application of anodically generated hypervalent iodine reagents in the presence of oxidatively resistant fluorous solvents (HO–R_F_) which act as ligands to form ArI(OR_F_)_2_ intermediates (Figure 5b). The latter approach has provided a demonstration of the viability of hypervalent iodine electrosynthesis but fails to address the central challenge in catalysis, which is selective catalyst oxidation in the presence of oxidatively labile substrates.^55−57^

**Figure 6:**
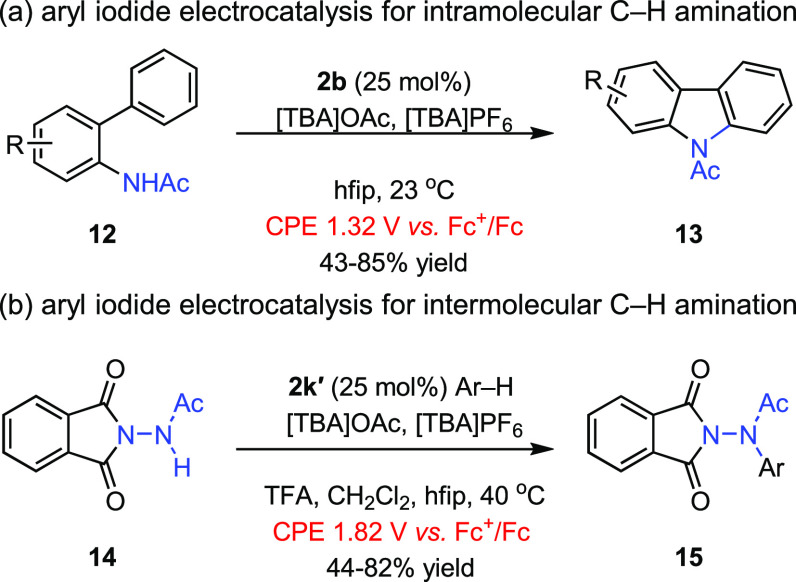
Aryl iodide electrocatalysis for (a) intra- and (b) intermolecular C–H amination is efficient in the presence of carboxylate sources (i.e., [TBA]OAc) added to stabilize iodanyl radical intermediates. **2k′**: 2,2′-diiodo-4,4′,6,6′-tetramethyl-1,1′-biphenyl. [TBA]: *tetra*-butyl ammonium. TFA: trifluoroacetic acid. hfip: 1,1,1,3,3,3-hexafluoroisopropanol.

Consistent with our hypothesis that carboxylate-stabilized iodanyl radicals are on-path for aerobic hypervalent iodine synthesis, our initial forays into aryl iodide electrocatalysis indicated a decisive role for carboxylate additives:^3^ Electrolysis of biaryl amide **12**, which was selected based on the seminal C–N bond-forming chemistry disclosed by Antonchick and co-workers,^58^ in the presence of various aryl iodides and [TBA]PF_6_ as a supporting electrolyte failed to deliver the desired carbazole product **13**. The addition of one equivalent of [TBA]OAc as part of the supporting electrolyte mixture resulted in efficient C–N bond construction. Similarly dichotomous results, in which catalysis was not observed in the absence of carboxylate but was efficient upon the addition of carboxylate additives, were obtained across a suite of carbazole-forming intramolecular C–N bond constructions (Figure 6a) as well as in related intermolecular C–H/N–H coupling (i.e., conversion of **14** to **15**, Figure 6b). In comparison to the original report by Antonchick and co-workers in which I(III) reagents were used to promote intramolecular C–N bond construction, the electrocatalytic carbazole formation protocol exhibited higher functional group tolerance with comparable yields.

The development of electrochemical conditions was based on the hypothesis that interrupted Kolbe electrochemistry would provide acetoxy radicals, which would ultimately afford hypervalent I(III) compounds similar to aerobic oxidation chemistry. Subsequent electrochemical studies are instead most consistent with initial oxidation of the aryl iodide: Strong hydrogen-bonding between hfip and acetate suppresses direct acetate oxidation and prevents Kolbe electrochemistry at the potentials utilized for electrocatalysis.

The results of this initial foray into hypervalent iodine electrocatalysis resulted in two tantalizing conclusions. First, because the primary anodic oxidation event removes an electron from the aryl iodide and not acetate, the structure of the aryl iodide can be used as a tool to control the potential at which catalysis proceeds.^59^ Second, in the absence of biaryl amide **12**, electrolysis of **2b** did not result in the formation of I(III) species; rather, bulk electrolysis of **2b** resulted in deiodinative coupling to afford 4,4′-dimethoxy-1,1′-biphenyl.^59^ Together, these observations raised the unexpected prospect that iodanyl radicals, and not I(III) intermediates, may be on-path for substrate functionalization.

**Figure 7:**
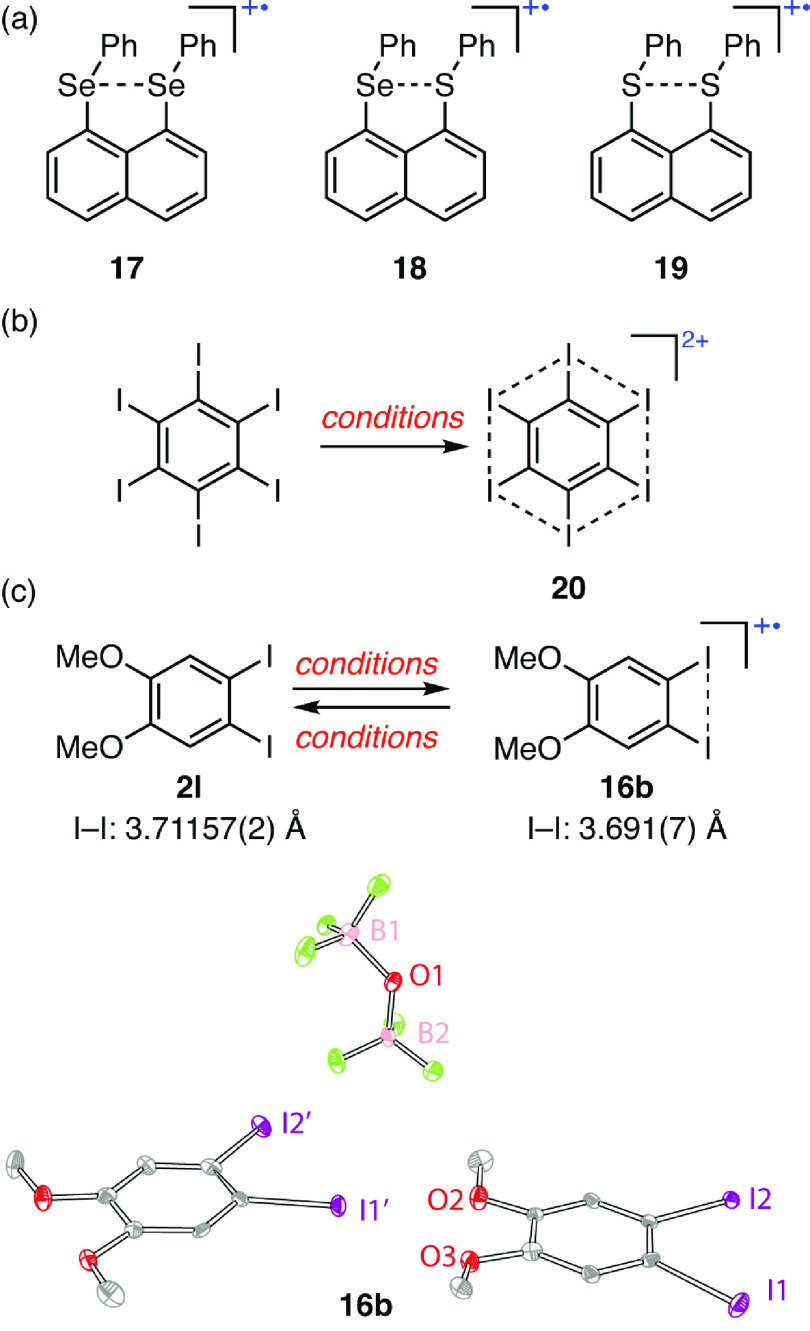
(a) Examples of oxidatively induced bonding in heavy main group compounds. (b) Treatment of hexaiodobenzene with either Cl_2_ or H_2_O_2_ in triflic acid yields a singlet dication (**20**) with delocalized σ and π bonding. (c) Displacement ellipsoid plot of **16b** generated by chemical oxidation of **2l** with 0.5 equiv of PIFA and excess BF_3_·OEt_2_, in CH_2_Cl_2_ at −22 °C.

As we considered strategies to lower the onset potential for anodic oxidation of aryl iodides, we were inspired by reports of oxidatively induced partial bonding in main group compounds. For example, oxidatively induced bonding has been reported for 1,8-disubstituted naphthalenes with proximal selenium and sulfur substituents (i.e., **17**–**19**, Figure 7a).^60−63^ Additional inspiration came from the report that 2-fold oxidation of hexaiodobenzene results in *bis-*cation **20**, which is aromatic in both the σ and π frameworks (Figure 7b).^62^ Based on the hypothesis that oxidation-induced I–I bonding interactions could facilitate anodic aryl iodide oxidation, we identified 1,2-diiodo-4,5-dimethoxybenzene **2l** as a highly efficient catalyst for C–H/N–H coupling capable of operating at as low as 0.5 mol % loading. Compound **2l** displays a highly reversible one-electron oxidation feature by cyclic voltammetry (*E*_1/2_ = 1.19 V vs Fc^+^/Fc, *I*_p_ = 0.94), and the applied potential for efficient catalysis was 100 mV lower than analogous catalysis with 4-iodoanisole (**2b**).^4^ The electrochemical data observed is coincident with the one-electron oxidation of **2l** to **16b**, which raised the possibility that substrate activation proceeds from an open-shell intermediate without the intermediacy of I(III) species.

Electrolysis of **2l** in the absence of a substrate does not result in any observable I(III) species by ^1^H NMR; rather, it results in the formation of a dark blue solution. UV–vis spectroscopic experiments coupled with time-dependent DFT (TD-DFT) calculations were consistent with the anodic generation of iodanyl radical **16b**. *In situ* EPR spectroscopy during electrolysis of **2l** in TBAPF_6_/hfip supported the formation of an open shell intermediate. Natural transition orbital (NTO) analysis suggests significant spin density on the iodine atoms. Crystallization of **16b** enabled characterization by single-crystal X-ray diffraction and revealed significant contraction of the I–I distance upon one-electron oxidation, which is consistent with the formation of a two-centered, three-electron (2c–3e) σ bond (Figure 7c).^61^

## 5 Elementary Steps in Iodanyl Radical Chemistry

### Iodanyl Radicals in Bidirectional PCET

Formally I(II) compounds are rare species in organic chemistry. The potential intermediacy of iodanyl radicals has been suggested in a number of contexts. Kita and co-workers demonstrated one-electron oxidation of electron-rich arenes with hypervalent iodine(III) reagents to produce arene radical cations.^64^ Redox balance would suggest the coevolution of undetected iodanyl radicals under these conditions. Subsequently, Maruoka et al. proposed the intermediacy of iodanyl radicals in the context of photochemical C–H halogenation reactions. The noted importance of aryl iodide structure on site selectivity was suggested to be evidence that iodanyl radical intermediates were involved in hydrogen atom abstraction chemistry.^65^ More recently, Studer and co-workers utilized hypervalent iodine(III) compounds as radical precursors for C–H functionalization chemistry.^37^

The development of (electro)chemical conditions to access iodanyl radical **16b** provided the first opportunity to evaluate the elementary chemical steps that characterize the reactivity of iodanyl radicals and evaluate the chemical competence of such an open-shell intermediate to effect bond-forming chemistry traditionally ascribed to I(III) species: Under conditions relevant to catalysis—biaryl amide **12** and [TBA]OAc in hfip solvent—isolated iodanyl radical **16b** is competent in the activation of **12** to afford carbazole **13**.

**Figure 8:**
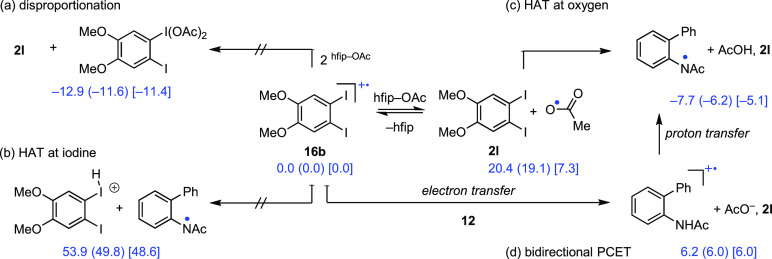
Summary of mechanistic pathways and thermodynamic calculations for possible iodanyl radical reactions, including (a) iodanyl radical disproportionation, (b) H-atom transfer to iodine, (c) acetate oxidation followed by H-atom transfer to oxygen, and (d) PCET pathways were considered. Computations use the UB3LYP/DGDZVP2-D3-SMD(2-methyl-1-propanol) level of theory, Δ*E* (Δ*H*) [Δ*G*].

We envisioned several reaction pathways by which the initially generated iodanyl radical cation could enact substrate functionalization including (1) ultimate formation of I(III) species via disproportionation (Figure 8a),^2^ (2) H-atom transfer (HAT) at iodine (Figure 8b),^66−68^ (3) acetate oxidation and HAT at the acetoxy radical (Figure 8c),^69^ or (4) a proton-coupled electron transfer (PCET, Figure 8d).^4^ Available experimental and computational data are most consistent with activation of the N–H valence of **12** by bidirectional PCET reaction in which the carboxylate additive accepts the proton and the iodanyl radical accepts the electron (Figure 8d). A second PCET step is needed from aminyl radical **17** to generate the observed products, and this step was calculated to be substantially exothermic (−79.7 kcal/mol). Comparison of the computed thermodynamics of acetate binding to the radical cation of iodoanisole (**2b**, −2.8 kcal/mol) and **16b**^**+**^ (3.2 kcal/mol) demonstrate that carboxylate binding to iodanyl radical cations is highly dependent on aryl iodide structure and thus whether the iodanyl radical engages the substrate as a carboxylate adduct or as a charge-separated ion pair is likely catalyst dependent.

### Disproportionation of Iodanyl Radicals

**Figure 9:**
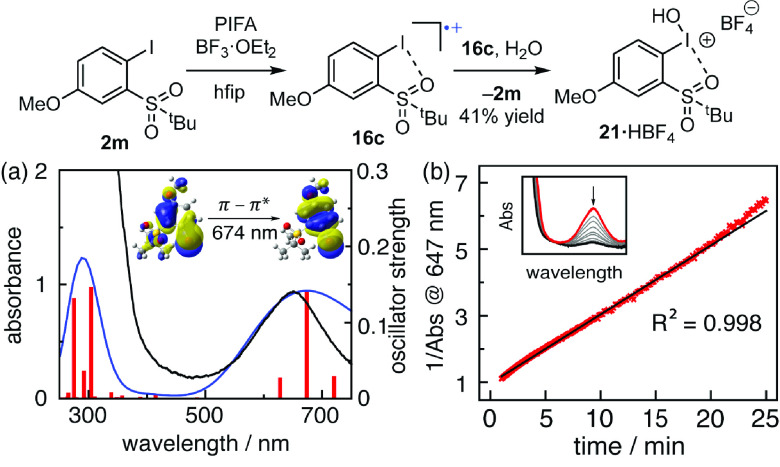
Iodanyl radical disproportionation. (a) UV–vis spectroscopy of **2m** treated with 0.5 equiv PIFA and BF_3_·OEt_2_ in hfip (black), time-dependent density functional theory (TD-DFT) of computed **16c** (blue), and electronic configurations for excited state **16c** (red). Inset: the highest occupied transition orbital (HOTO) to lowest unoccupied transition orbital (LUTO). (b) Plot of 1/Abs_647_ vs time providing a second-order dependence on **16c** decay. hfip: 1,1,1,3,3,3-hexafluoroisopropanol.

We proposed disproportionation of the iodanyl radical to I(I) and I(III) compounds as one of the termination steps in the aldehyde autoxidation-promoted oxidation of aryl iodides without experimental validation.^2^ During electrosynthetic studies of I(III) compounds featuring *ortho*-Lewis basic substituents, which Protasiewicz introduced to enhance the solubility of iodosyl- and iodoxybenzenes,^70^ we had the opportunity to experimentally validate I(II) disproportionation as an elementary step characteristic of I(II) compounds (Figure 9).^71^

Chemical oxidation of **2m** by PIFA and BF_3_·OEt_2_ in hfip, conditions previously developed to prepare iodanyl radicals,^4^ resulted in the immediate formation of a dark blue solution with λ_max_ = 647 nm (Figure 9a). The observed spectral features were well-reproduced by TD-DFT calculations of iodanyl radical **16c** (Figure 9b). The decay of the spectral features of **16c** follows second-order kinetics, and following complete bleaching of the blue color, I(I) species **2m** and I(III) species **21·HBF**_**4**_ were observed by ^1^H NMR spectroscopy. These results confirm the decomposition of iodanyl radical **16c** by disproportionation.

## 6 Sustainable Iodoxybenzene Chemistry

In addition to the aforementioned chemistry of organoiodine(III) compounds, there is a rich chemistry available to the higher-valent organoiodine(V) reagents. The chemoselectivity of I(III)- and I(V)-based reagents is complementary, with I(III)-based reagents engaging in group-transfer and olefin functionalization chemistry while I(V)-based reagents typically engage in dehydrogenative chemistry of alcohol and amine substrates. We were attracted to sustainable synthetic methods to generate I(V) species as a platform to expand the sustainable synthetic chemistry available from iodanyl radical intermediates.

**Figure 10:**
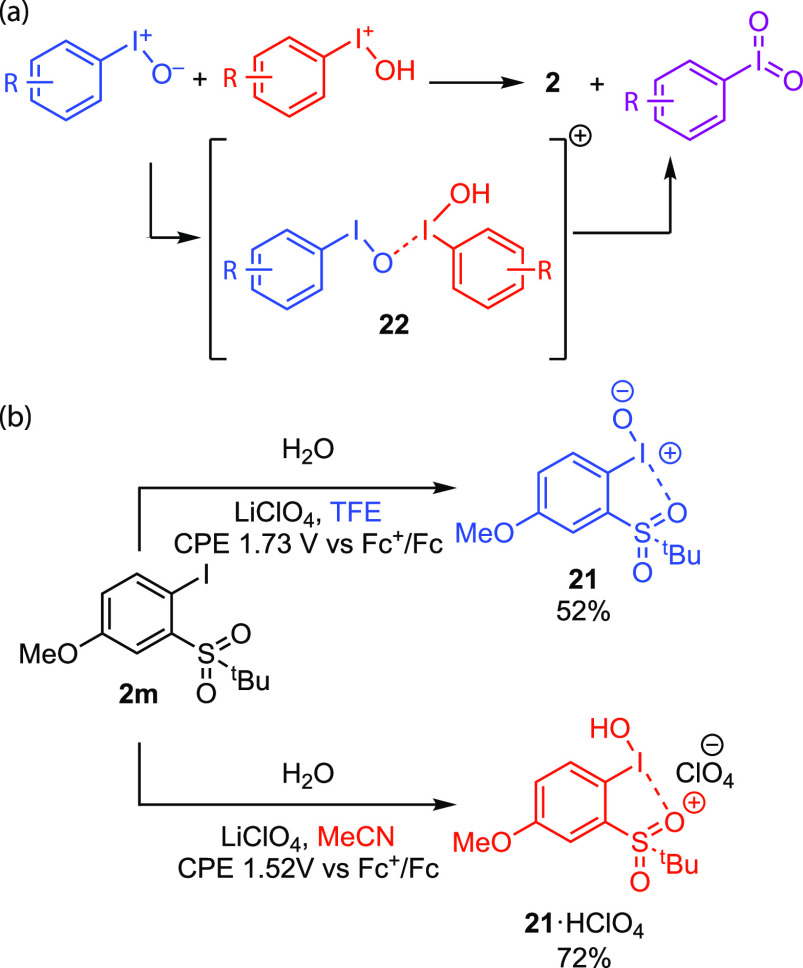
(a) Koser suggested the intermediacy of an *O*-bridged diiodide in the disproportionation of iodosylbenzenes. (b) Solvent dependence on the electrosynthesis of protonated and unprotonated iodosylbenzene from **1n**.

Our efforts in sustainable I(V) chemistry have been motivated by the fact that I(III) compounds are often metastable with respect to disproportionation to I(I) and I(V) reagents. Despite the thermodynamics of this disproportionation, significant kinetic barriers typically prevent facile disproportionation, and thus catalysts and/or high temperatures are needed to promote disproportionation.^72^ For example, in 1997 Koser reported a detailed study of the acid-promoted disproportionation of iodosylbenzene and proposed that disproportionation proceeds through an unobserved *O*-bridged intermediate (**22**) that is generated by a combination of a molecule of iodosylbenzene with a protonated iodosylbenzene (Figure 10a).^73^ As a result of the typically slow disproportionation kinetics, I(V) electrosynthesis is accomplished by serial oxidation of I(I) to I(III) and subsequent oxidation of I(III) to I(V) under more forcing conditions. We reasoned that development of facile I(III) disproportionation chemistry would enable I(V) chemistry to be accessed with the reagents and protocols already developed for sustainable I(III) chemistry and provide the mechanistic basis for I(V) catalysis.

During exploratory electrosynthetic studies based on the disproportionation of anodically generated iodanyl radical **16c**, we identified solvent-dependent reaction outcomes, with **21·H**^**+**^ being generated in MeCN and **21** being generated in TFE (Figure 10b).^71^ Consistent with the Koser hypothesis for iodosylbenzene disproportionation, the combination of these two protonation states under ambient conditions results in rapid disproportionation to I(I) and I(V). Combination at low temperatures enabled the critical *O*-bridged intermediates to be isolated and crystallographically characterized (Figure 10c). Implementing these insights in the context of a direct electrolysis of **2m** in a mixed solvent system resulted in the direct electrosynthesis of I(V) compounds. Critically, by leveraging sequential disproportionation, first via the disproportionation of sustainably generated I(II) intermediates to gain entry to I(III) compounds and then by controlled disproportionation of those I(III) compounds to access I(V) compounds, we avoid the need for more forcing conditions to achieve serial oxidation and provide the opportunity to couple sustainably generated iodoxybenzenes to substrate functionalization.

**Figure 11:**
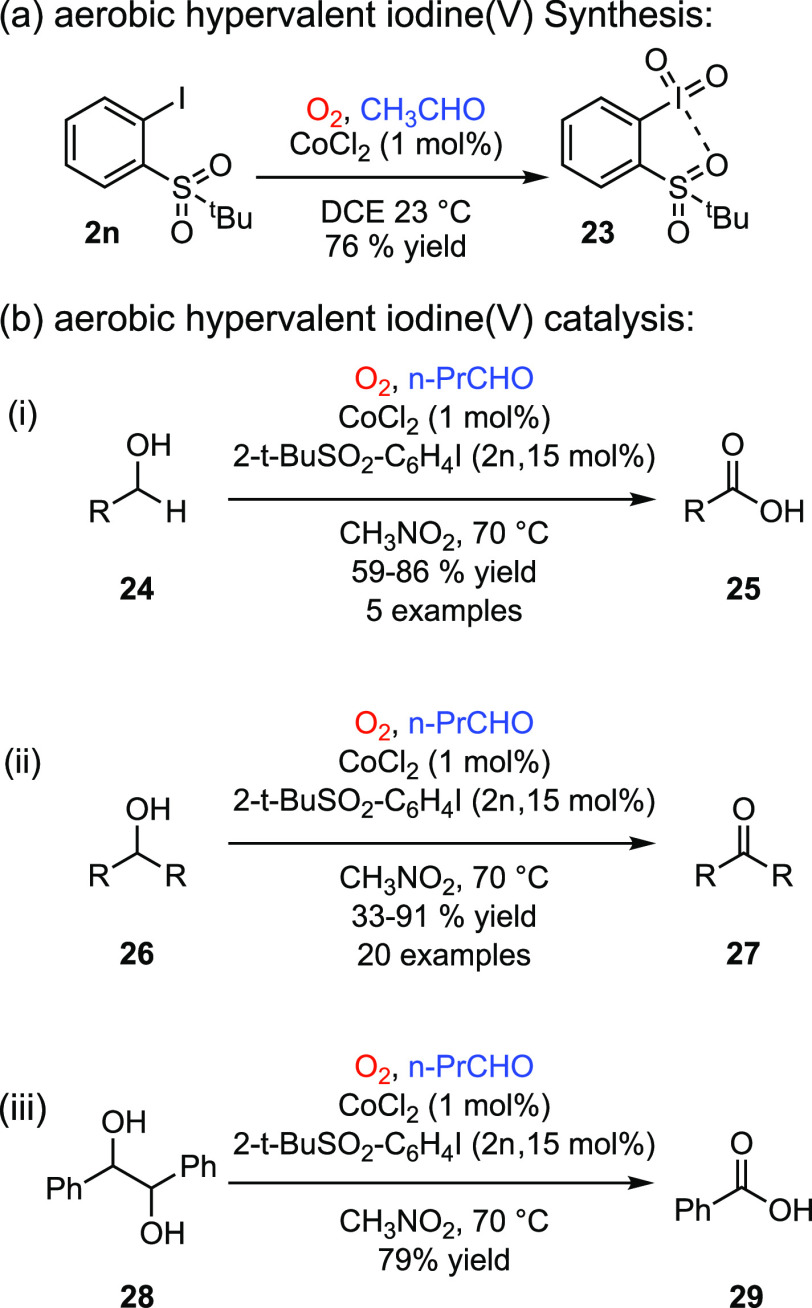
(a) Aldehyde autoxidation-interrupted synthesis of I(V) derivative **23**. (b) Aerobic oxidation catalysis with **2n** includes (i) primary alcohol oxidation to carboxylic acids, (ii) secondary alcohol oxidation to ketones, and (iii) 1,2-diol cleavage. DCE: 1,2-dichloroethane.

Similarly, *tert*-butyl sulfonyl substituted iodosylbenzenes also display aerobic iodoxybenzene chemistry. During early studies of the aerobic synthesis of hypervalent iodine compounds, we found that 2*-tert*-butylsulfonyl iodobenzene (**2n**) resulted in the corresponding I(V) compound (**22**) instead of the anticipated I(III) compound (Figure 11a).^74^ Exposure of independently synthesized I(III) compounds, derived from **2n**, to the autoxidation conditions led to rapid disproportionation, and the facility of this chemistry enabled aerobic oxidation catalysis of reactions characteristic of Dess-Martin periodinane, such as oxidation of alcohols (**23**) to carboxylic acids (**24**) or ketones (**25**), and oxidative cleavage of 1,2-diols (**27**, Figure 11b).^75,76^ The rapidity of disproportionation under the aerobic conditions complicated interrogation of the mechanism of disproportionation chemistry in this system.

## 7 Conclusion and Perspective

This Account has described the development of a research program in sustainable hypervalent iodine chemistry. Motivated by the two-electron oxidation and reduction processes that characterize the reactivity of hypervalent iodine compounds, we targeted these molecules as a platform to merge the one-electron reactions of sustainable oxidation with the two-electron transformations of selective synthetic chemistry. While our original hypothesis led to the identification of efficient aerobic oxidation conditions based on interrupted aldehyde autoxidation, the hypothesis did not withstand experimental scrutiny. Detailed experimental and computational studies revealed an unexpected role for iodanyl radical intermediates in the aerobic synthesis of hypervalent iodine compounds.

The central role of iodanyl radicals in aerobic hypervalent iodine chemistry led to the development of new strategies in electrocatalysis. These efforts were predicated on harnessing anodically generated iodanyl radicals for hypervalent iodine electrosynthesis. This program in electrocatalysis also led to new strategies to synthesize, stabilize, and isolate formally I(II) compounds and to evaluate the elementary chemical steps available to these compounds. Both disproportionation and substrate activation via PCET mechanisms have been documented, and additional modes of reactivity may remain to be discovered. Furthermore, the demonstration that isolable iodanyl radicals, and not downstream I(III) derivatives, can engage in substrate functionalization opens new opportunities for redox catalysis via open-shell aryl iodide derivatives, which may display complementary chemoselectivity vis-à-vis I(III) intermediates.

More broadly, open-shell P(IV)^77^ and Bi(II)^25^ intermediates have garnered interest in the past few years as intermediates in a variety of catalytic protocols. Together, these reports suggest a broader role for heavy main-group radicals in catalysis than had previously been understood. Ongoing studies to identify unique modes of substrate engagement at open-shell intermediates promise to expand the opportunities for catalysis at heavy main group radicals, and the development of strategies to stabilize open-shell compounds will both enable the study of these exotic species and enable new metal-free catalysis.
